# Global neurosurgery amongst the EANS community: Where are we at?

**DOI:** 10.1016/j.bas.2022.100911

**Published:** 2022-06-28

**Authors:** Nicolò Marchesini, Marcel Ivanov, Jesus Lafuente, Francesco Sala, Nikos Foroglou, Massimiliano Visocchi, Fatos Olldashi, Pablo Gonzalez-Lopez, Jamil Rzaev, Magnus Tisell, Vincenzo Paternò, Kresimir Rotim, Jake Timothy, Lukas Rasulic, Andreas K. Demetriades

**Affiliations:** a Global and Humanitarian Neurosurgery Committee, European Association of Neurosurgical Societies (EANS), UK; b Department of Neurosurgery, University Hospital Borgo Trento, Verona, Italy; c Department of Neurosurgery, Sheffield Teaching Hospital, NHS Foundation Trust, UK; d Spine Center, Hospital Del Mar, Barcelona, Spain; e Department of Neurosurgery, AHEPA University Hospital, Medical School, Aristotle University of Thessaloniki, Greece; f Institute of Neurosurgery, Catholic University of Rome, Italy; g Department of Neurosurgery, University Hospital of Trauma, Tirana, Albania; h Department of Neurosurgery, General University Hospital Alicante, Alicante, Spain; i Federal Neurosurgical Center, Novosibirsk, Russian Federation; j Department of Neurosurgery, Sahlgrenska University Hospital, Göteborg, Sweden; k Department of Neurosurgery, International Neuroscience Institute, Hannover, Germany; l Department of Neurosurgery, University Hospital Sisters of Mercy, Zagreb, Croatia; m Department of Neurosurgery, Leeds General Infirmary, Leeds, UK; n Faculty of Medicine, University of Belgrade, Clinic for Neurosurgery, University Clinical Center of Serbia, Belgrade, Serbia; o Department of Neurosurgery, Royal Infirmary, Edinburgh, UK

**Keywords:** **HICs**, high-income countries, **LMIC**, low- and middle-income countries, **EANS**, European Association of Neurosurgical Societies

## Abstract

• Awareness of Global Neurosurgery opportunities is limited in the EANS and a minority have had previous experiences with such activities.
• Most training programs and job environments don't encourage participation in Global Neurosurgery and mentors are lacking.
• However, most European neurosurgeons and trainees remain interested in Global Neurosurgery and are willing to participate.
• Junior trainees is the group with the highest rate of interest for Global Neurosurgery.
• Barriers exist that may limit participation in Global Neurosurgery, and funding is the most relevant.

## 1 Introduction

The gap between HICs and LMICs in the capability of fulfilling the surgical needs of their population has been fully recognized and quantified in a recent Lancet Commission on Global Surgery (Meara et al., 2015). As regards Neurosurgery specifically, approximately five million people annually are estimated to be left untreated in LMICs for essential neurosurgical conditions that would elsewhere undergo surgical intervention (Dewan et al., 2018). The acquisition of awareness that surgical treatments, including neurosurgical, are an “indivisible and indispensable” component of health, is leading to a series of initiatives aiming to reduce these gaps amongst the different realities (Farmer and Kim, 2008; Johnson, 2013; Ozgediz et al., 2005; J. K; World Health Organization, 2015).

Global Neurosurgery, with its primary purpose of “ensuring timely, safe and affordable neurosurgical care to all who need it” well fits in this so-called “the surgery spring” (Mullan, 2015; Rosseau et al., 2020). Global Neurosurgery initiatives have now broadened their spectrum of action from the initial pioneering surgical camps to provide free surgical care to those in need, which maintain an essential role. These initiatives now include a series of clinical, research and educational activities that go beyond “surgery” strictly speaking. These are represented by, but are not limited to, providing education to local actors, addressing specific issues within the health systems, and developing visiting residencies or fellowships for bilateral neurosurgical transfer of knowledge and competencies (Haglund and Fuller, 2019).

The EANS is a vibrant community that shares the values expressed in the preamble of the Constitution of the World Health Organization which states that “the enjoyment of the highest attainable standard of health is one of the fundamental rights of every human being without distinction of race, religion, political belief, economic or social condition” (Constitution of the World Health Organization, 1946). Keeping this in mind, the EANS is making all possible efforts to put Global Neurosurgery initiatives amongst the priorities of its agenda. With the commencement of its term of Office in October 2021, the 2021–2023 Board of the EANS, as one of its first actions, approved the formation of a *Global and Humanitarian Neurosurgery Committee.*

With the aim to better explore energies, efforts and resources, the EANS conducted this survey, with the specific purpose to map awareness, interest and barriers for Global Neurosurgery development amongst its members.

## 2 Methods

An electronic online survey was developed using Google Modules (Google©). Questions were structured to explore general demographics, previous experiences, awareness, interests and possible barriers that may limit participation in Global Neurosurgery activities. Questions were, in part, analogous to what had previously been done by others (Westwick et al., 2020). Lickert scales were used to assess the rate of agreement with specific sentences. Barriers and motivating factors for participating in Global Neurosurgery rotations were asked to be graded from 0 (no important at all) to 3 (very important).

The questionnaire was targeted at Neurosurgeons and trainees in Neurosurgery specialised or in-training in one of the EANS countries (EANS member Societies, 2021). Between June 6th^,^ 2021 and December 15th^,^ 2021 the survey was spread by social media (Facebook, WhatsApp, Telegram, Instagram, Twitter) and the EANS official website and newsletter. The survey was also promoted during three EANS Training Courses within 2021 (Valencia Spring, 2021; Thessaloniki Summer, 2021; Valencia Autumn, 2021). Due to wide dissemination by social media, the calculation of a response rate was not possible.

Data were prospectively collected and the results were tabulated in a Microsoft Excel spreadsheet. Pearson's chi-square test was used to assess measures of association in frequency tables. Statistical analysis was performed by commercially available software (Stata version 16.1). For statistical significance values of p ​< ​0.05 were considered.

The order in which results are presented in the next section does not necessarily follow the order of the questions in the questionnaire (Annex I).

## 3 Results

### 3.1 Demographics and previous experiences

**Fig. 1:**
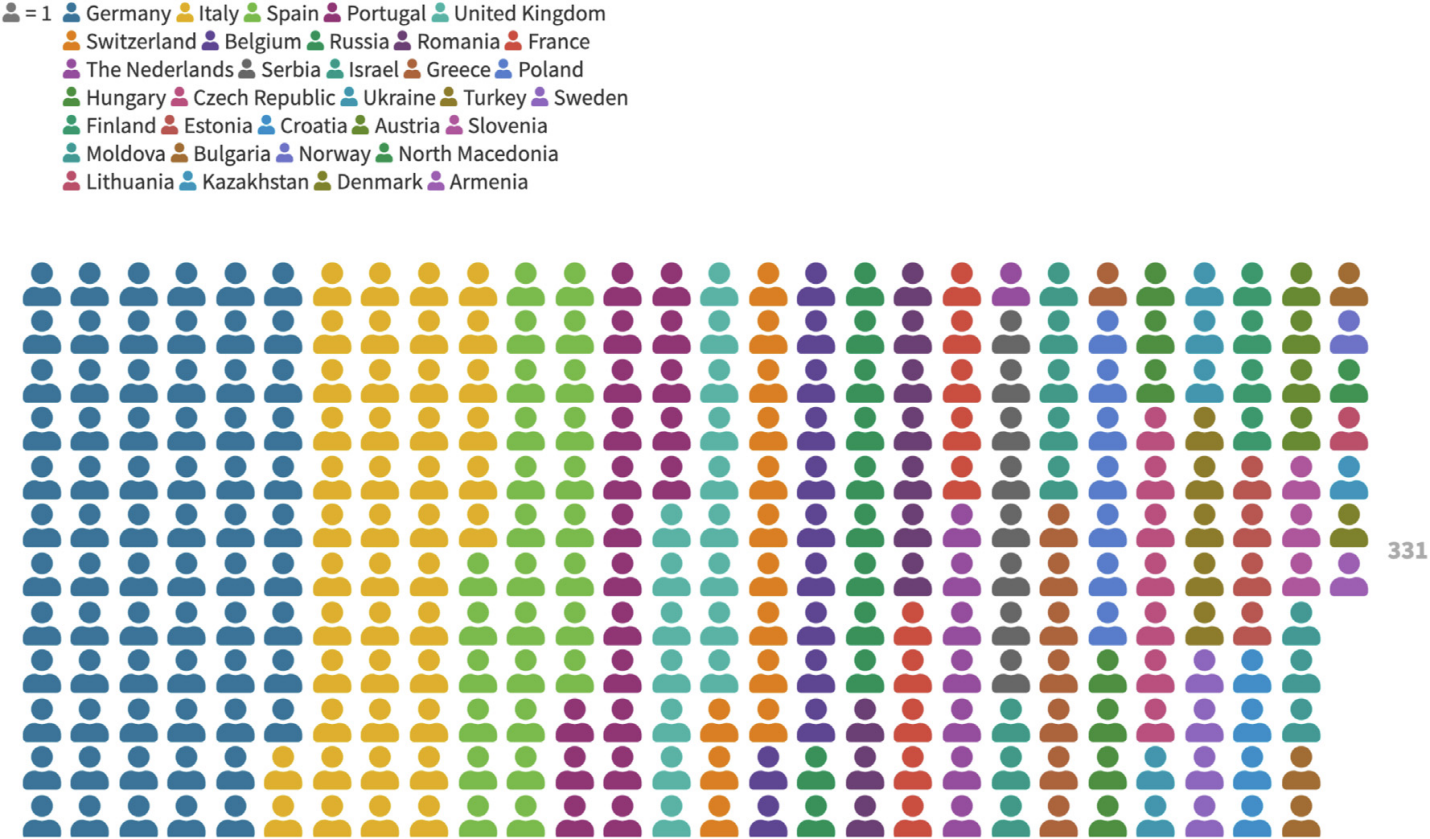
Detailed geographical distribution of the 331 respondents.

Overall, 331 responses were obtained. All but six respondents (98.2%) were practising in one of the EANS countries at the time of survey administration. The answers came from 33 of the 39 EANS member societies (84.6%) (see Fig. 1). The country with the largest number of answers was Germany (70; 21.1%) and no answers were obtained from Albania, Bosnia, Cyprus, Kosovo, Latvia and Slovakia. Most respondents were aged between 30 and 34 (142; 42.9%) and most were engaged in a relationship (215; 65%). The majority of answers came from trainees (169; 51.1%) and most of the respondents were officially EANS subscribed members (255; 77%). Nearly 60% had previous academic abroad experiences during medical school years (e.g. Erasmus program), and 39% had non-academic experiences (e.g. volunteering).

**Fig. 2:**
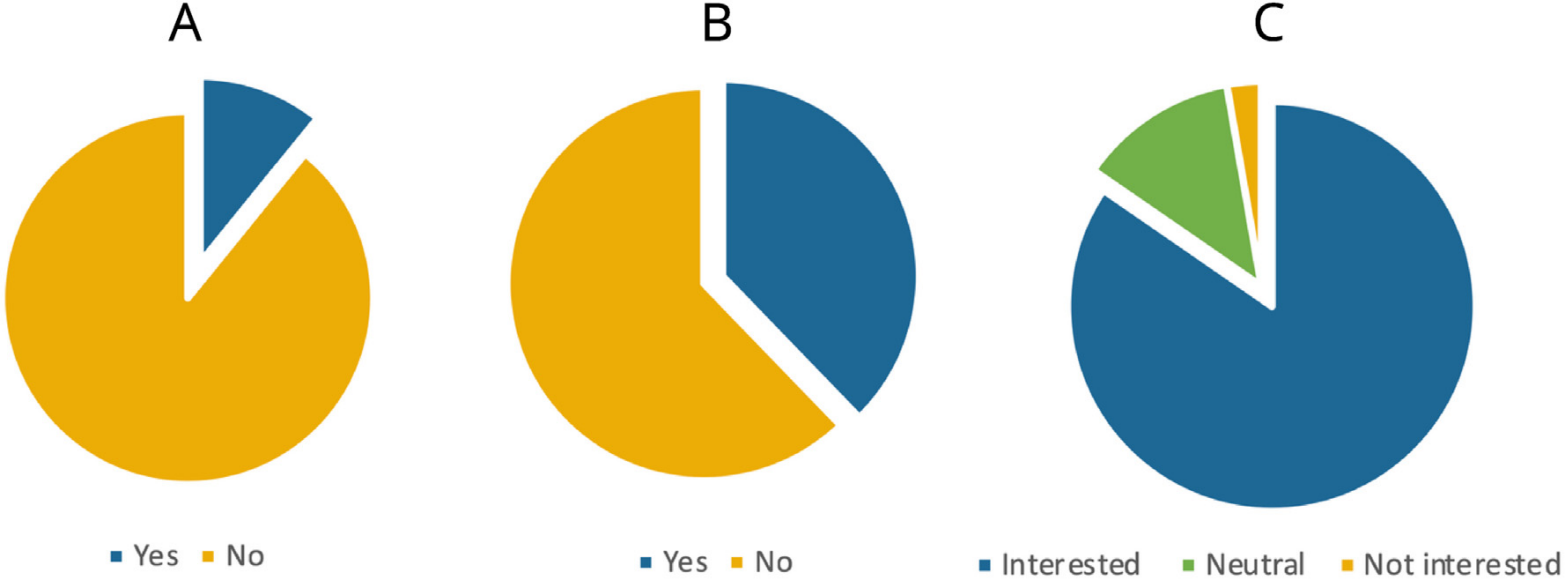
Proportion of respondents reporting (A) previous experiences with Global Neurosurgery rotations, (B) actual awareness of Global Neurosurgery opportunities for residents/neurosurgeons, (C) interest in participation in Global Neurosurgery rotations.

Specifically on Global Neurosurgery rotations, only 36 (10.9%) stated to have participated in such an experience during Residency/Training (see Fig. 2). Overall, only 6.3% (21/331) had travelled to regions within LMICs. The most common length of stay of neurosurgical rotation was one to six months (18; 50%) while six respondents (16.7%) stayed for less than one month. Demographic data are summarized in Table 1.

**Table 1:** Demographics and general information of the 331 respondents.

Variable	Number (%)
Respondents	331 (100)
Age	
<25	3 (0.9)
25–29	55 (16.6)
30–34	142 (42.9)
35–39	58 (17.5)
50–59	68 (20.5)
>60	5 (1.5)
Marital status	
Married/committed	215 (65)
Single	112 (33.8)
Not answering	4 (1.2)
Surgical status	
Junior trainee	47 (14.2)
Senior trainee	122 (36.9)
Fellow	23 (6.9)
Specialist	133 (40.2)
EANS membership	
Yes	255 (77)
No	76 (23)
Medical school academic abroad experiences	
Yes	198 (59.8)
No	133 (40.2)
Medical school non-academic abroad experiences	
Yes	129 (39)
No	202 (61)
Global Neurosurgery experiences during Residency	
Yes	36 (10.9)
No	295 (89.1)

### 3.2 Awareness

Overall, only 37.8% (125/331) of the respondents were aware of Global Neurosurgery opportunities for neurosurgeons and trainees (see Fig. 2). The professional groups with the highest rate of awareness were junior trainees (23/47; 48.9%) and specialists (61/133; 45.9%) against 26.2% of senior trainees (32/122) (p ​= ​0.0048 and p ​= ​0.0011). EANS individual members stated awareness in 36.1% of cases (92/255).

One hundred and twenty-three respondents stated to follow Global Neurosurgery developments and updates (123; 37.2%). These were 38.3% of junior trainees (18/47), 23% of senior trainees (28/122), 73.9% of fellows (17/23) and 45.1% of specialists (60/133). Significant differences were found between senior trainees and junior trainees (p ​= ​0.0446), fellows (p ​< ​0.0001) and specialists (p ​= ​0.0002).

The presence of a faculty member involved in Global Neurosurgery projects within a respondent's Department was reported by 23.3% (77/331).

### 3.3 Interest in global neurosurgery

Overall, two hundred and eighty respondents (84.6%) stated to be interested (agree or strongly agree) in participating in Global Neurosurgery rotations while nine (2.7%) were not interested (disagree or strongly disagree) (see Fig. 2). The rate of interest was 91.5% amongst junior trainees (43/47), 79.5% amongst senior trainees (97/122), 89,7% amongst fellows (26/29), and 85.7% amongst specialists (114/133). These differences were not significant (p ​> ​0.05). Moreover, there were no significant differences in the rate of interest between committed and single respondents (87.5% vs 82.8%), EANS and non-EANS members (84.3% vs 85.5%), having participated or not in academic and non-academic abroad experiences during medical school (83.8% vs 85.7%; 88.4% vs 82.2%). The rate of interest was significantly higher in those who answered to follow Global Neurosurgery developments and updates (91.9% vs 80.3%; p ​= ​0.0048).

**Fig. 3:**
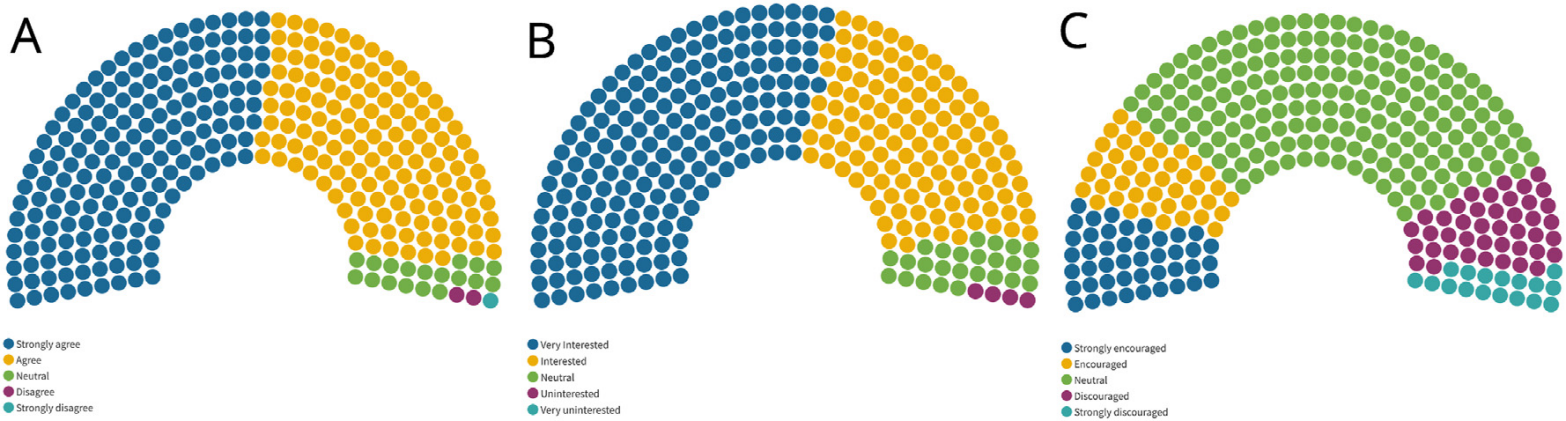
Rate of agreement with the question (A) whether neurosurgeons and trainees in neurosurgery could have a positive local impact during rotations abroad; and (B) whether there would be interest if a structured Global Neurosurgery program were available. Panel (C) explores the rate of encouragement in participation in Global Neurosurgery initiatives by the residency programs/job environment. Each point represents one respondent.

Of all respondents, 93.7% (310/331) believed they could have a positive local impact during Global Neurosurgery rotations abroad while only three disagreed (0.9%) (see Fig. 3).

The majority agreed or strongly agreed that Global Neurosurgery should be considered as a specific career track (148 and 126; 82.8%), not inferior to other potential career tracks like neurosurgeon-educator or neurosurgeon-scientist. Only eight disagreed or strongly disagreed (7 and 1; 2.1% and 0.3%). Similarly, most stated that increasing access to neurosurgical care in low and middle-income countries is a priority they would focus on in their career (212; 64%) and a small minority disagreed or strongly disagreed (29 and 6; 8.8% and 1.8%).

Most respondents would be planning to participate in Global Neurosurgery rotations as Neurosurgeons (210; 63.4%). Fellows reported the highest rate of agreement with this statement (19/23; 82.6%), significantly higher (p ​= ​0.027) when compared to senior trainees (71/122; 58.2%). Similar results were observed for single vs respondents engaged in a relationship (75.9% vs 56.3%; p ​= ​0.0005).

A vast majority (264/331; 79.8%) would be ready to self-fund their Global Neurosurgery rotation (186/264 partially, 70.5%; 78/264 completely, 29.5%) and only a minority would not use their vacation for this purpose (49; 14.8%). The ideal duration of a Global Neurosurgery experience abroad would be from one-to-six months for 48.9% of respondents and two-to-four weeks for 31.1%. The remaining would prefer longer or shorter stays.

The type of desired involvement included clinical activity with surgical exposure (287; 88.3%) teaching (163; 50.2%), research (111; 34.2%) observation (36; 11.1%) and clinical activity without surgical exposure (32; 9.8%).

The geographical regions in which the majority of the respondents stated a desire to rotate were East Asia and Pacific, Latin America and Caribbean (173 each; 53.9%) and Sub-Saharan Africa (155; 48.3%).

The vast majority (302/331; 91.2%) were interested or very interest in Global Neurosurgery rotations if a structured program was available through training or employment. Only four (1.2%) were not interested, and no one was very uninterested (see Fig. 3).

Two hundred and two respondents (61%) agreed to leave their contact details for potential involvement in future initiatives of the EANS Global and Humanitarian Neurosurgery Committee.

### 3.4 Barriers and motivating factors and means to increase participation

Overall, only 18.4% of respondents (61/331) stated that their training program offers (or offered when in training) possible international opportunities. Junior trainees had the highest rates of positive answers (18/47; 38.3%) when compared with senior trainees (20/122; 16.4% p ​= ​0.0022), fellows (2/23; 8.7% p ​= ​0.0100) and specialists (21/133, 15.8% p ​= ​0.0013).

An encouragement (encouraged or strongly encouraged) in participation in a Global Neurosurgery rotation by the Training program or the employment environment was reported by 26% (86/331) while in most cases the attitude was neutral (186/331; 56.2%) (see Fig. 3). The group with the highest rate of encouragement was junior trainees (23/47; 48.9%) when compared with senior trainees (14/122; 11.5% p ​< ​0.001), fellows (8/23; 34.8% not significant) and specialists (41/133; 30.8% p ​= ​0.0258).

**Fig. 4:**
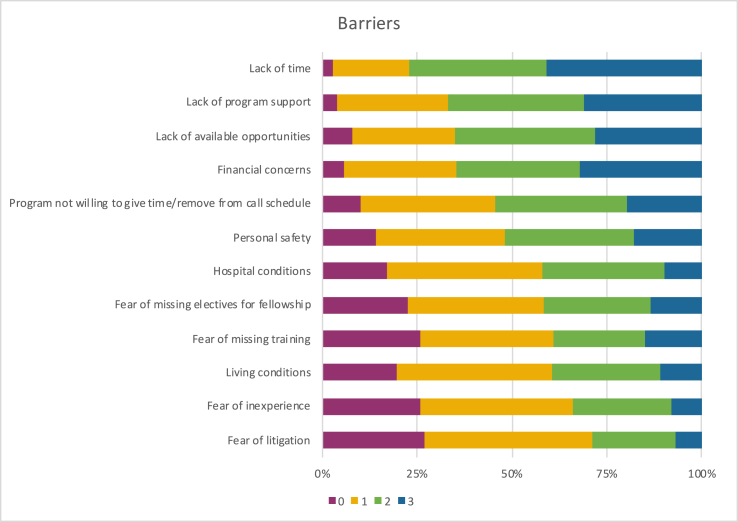
Possible barriers that could limit the participation in Global Neurosurgery initiatives (rating importance from 0 (not important at all) to 3 (very important).

Possible barriers limiting participation in Global Neurosurgery rotations with the highest degree of importance (2 and 3) included:

• lack of time (77%),
• lack of program support (67%),
• lack of available opportunities (65%) and
• financial concerns (65%) (see Fig. 4).

The least important (0 and 1) were fear of litigation (71%), fear of inexperience (66%), living conditions (61%) and fear of missing training (61%).

Lack of time was the most important limiting factor in all groups (79% junior trainees, 78% senior trainees and 71% specialists).

For junior trainees, this was followed by the program not being willing to give time or remove from on-calls (66%), financial concerns (60%) and lack of available opportunities (60%). For senior trainees, other relevant barriers were lack of training program support (74%) and lack of available opportunities (70%). For specialists, other relevant limiting barriers were financial concern, lack of support and lack of available opportunities (59% each).

The motivating factor with the highest degree of importance (2 and 3) was cultural experience (85%), the least important (0 and 1) was enhancing one's curriculum vitae (52%).

**Fig. 5:**
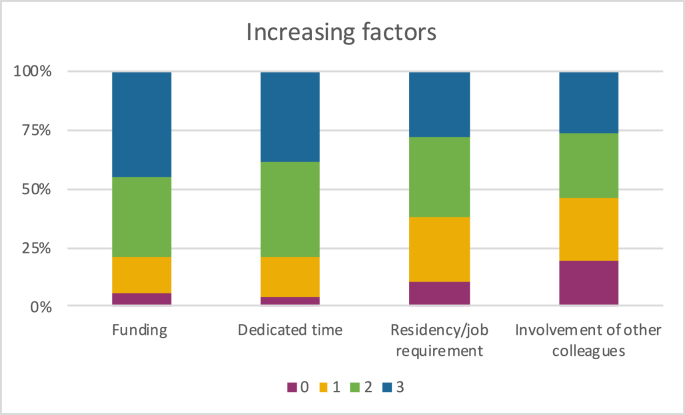
Possible factors that could increase the participation in Global Neurosurgery initiatives (rating importance from 0 (not important at all) to 3 (very important).

When asked what could be done to increase participation in Global Neurosurgery rotations, the factors with the highest degree of importance were funding opportunities (79%) and dedicated elective time for participation (79%) (see Fig. 5).

## 4 Discussion

Amongst the different types of Global Neurosurgery initiatives, rotations spent in LMICs by residents and specialists can constitute unique occasions for bilateral exchange and growth. From the clinical perspective, exposure to pathologies and patient care delivery in settings with limited resources can be an excellent occasion for improving personal knowledge and professional skills while contributing to the health administration in settings with high needs (Cadotte et al., 2014). In research, HICs professionals can assist global partners in creating local research infrastructures and sustainable projects. As funding and input from local governments and international policymakers constitute the basis for the development of neurosurgical care, visiting professionals can contribute by acting as effective health policy advocates (Lepard et al., 2020; Servadei et al., 2018).

Although the importance of Global Surgery initiatives has been recognized, only a few studies explored the rate of interest in such activities by HICs stakeholders (Powell et al., 2009; Butler et al., 2011; Cheung et al., 2018; Matar et al., 2012). As Neurosurgery represents a niche amongst the surgical specialties, studies investigating specific interests in Global Neurosurgery are even scarcer (Westwick et al., 2020; Davis et al., 2017; Mediratta et al., 2022). Filling this gap may be helpful to better address resources and energies in specific populations.

In a sample of 45 Canadian trainees in Neurosurgery, only 32% were aware of any Global Neurosurgery opportunities (Westwick et al., 2020). Altogether, European trainees seemed to have a very similar rate of awareness when compared to their Canadian counterparts (32.5%), even though a significant difference was found between junior and senior residents. Vice versa, European fellows and specialists had higher rates of awareness.

A previous survey, mostly based on fully trained European neurosurgeons, found that nearly 30% had travelled abroad as part of global neurosurgery initiatives. However, a significant proportion of HIC respondents indicated travels to North America as Global Neurosurgery activities. This could suggest a misinterpretation of the meaning of Global Neurosurgery in itself (Mediratta et al., 2022). This was found also in our sample, as of the 36 respondents that indicated previous Global Neurosurgery experience, only 21 had actually travelled to LMICs.

The low rate of participation in Global Neurosurgery rotations in LMICs in our sample (6.3%) seems to clash with the high rate of exposure to at least an undergraduate international experience (69.2%, 21.8% in LMICs), as such programs seemed to influence professional abroad choices after medical school (Żebryk et al., 2021). However, our results concerning previous Global Neurosurgery experiences were similar to those of analogous surveys (Westwick et al., 2020; Matar et al., 2012).

The value of mentorship in Neurosurgery goes beyond the transmission of mere technical skills, and mentor-mentee relation includes the transference of a series of core values that can influence the future of the new generations (Akhigbe et al., 2017). Global Neurosurgery is not different from other subspecialties in this, and the fact that less than one in four reported the presence of someone involved in such initiatives may hinder its development in European Institutions. In the Canadian study, nearly 60% of respondents reported the presence of at least one faculty with such interests within their department (Westwick et al., 2020).

Despite the seemingly low rate of awareness, previous experiences and environmental support, European neurosurgeons and trainees may represent an important potential source for Global Neurosurgery projects. Indeed, the stated interest in participating in Global Neurosurgery rotations was noteworthy for all the sub-groups of analysis, even higher than in other similar studies (Westwick et al., 2020).

The significantly higher interest in Global Neurosurgery rotations in those who follow Global Neurosurgery development and updates may be interpreted both as a cause and a consequence. Therefore, promoting the spread of Global Neurosurgery contents could be helpful to increase interest in participation as a virtuous circle.

The interest of European neurosurgeons in Global Neurosurgery is also formally confirmed by the high number of partnerships built with LMICs Institutions. When defining Global Neurosurgery as “any collaboration between a HIC and an LMIC to deliver, develop or study neurosurgical care in LMIC”, the HICs with most such collaborations seem to be represented by the USA and Canada. However, in the top ten HICs involved in Global Neurosurgery efforts, seven are represented by EANS nations (France, United Kingdom, Spain, Germany, Switzerland, Italy and Norway). (Fuller et al., 2021).

The disproportion in awareness, interest and willingness to participate between junior and senior trainees may be meaningful and can have several causes. The higher clinical and surgical demands, higher responsibilities and probably less time for academic activities may temporarily limit the available time and energies to dedicate to Global Neurosurgery for senior trainees. However, it seems that after residency completion, awareness and interest increase, with the new role of fellow and specialist. This may outline the importance that structured opportunities during formal training may have in maintaining and possibly energising the interest in Global Neurosurgery. Other medical specialties include in their curricula formal global health education initiatives and allow trainees to spend rotations in LMICs, but for surgical specialties the number of such initiatives is lower (Knudson et al., 2015; Hau et al., 2017).

In USA, among 115 neurosurgical residency/training programs, 28 (24%) offer documented global health opportunities (Rolle et al., 2020). In contrast, less than one in five respondents from our survey stated to have or have had international opportunities in LMICs during training. However, the fact that this proportion was significantly higher for junior residents (38.3%) and that a more encouraging environment seems to be available for this category (48.9%) could mean that something is changing. In a previous survey on European Neurosurgeons, 87% agreed that Global Neurosurgery rotations should be incorporated in training programmes (Mediratta et al., 2022).

Lack of time and financial concerns were universally stated as the main barriers limiting participation in Global Neurosurgery rotations in our study and the same results were found in previous ones (Westwick et al., 2020; Matar et al., 2012; Mediratta et al., 2022). It is meaningful that the majority of respondents would use their vacation time and would at least partially finance their stay in order to participate in Global Neurosurgery rotations. This strongly correlates with the belief in the potentially positive local impact that neurosurgeons and trainees may have abroad, as stated by more than 90% of respondents. However, the addition of dedicated time within training programs and job environments and the search for new ways of funding such initiatives is desirable, as these were stated as the main factors that could increase participation (Fernando et al., 2017).

In October 2021, the EANS Board published within its bylaws the constitution of the *Global and Humanitarian Neurosurgery Committee* as a successor of the previous International Relations Committee (The EANS Bylaws, 2021). The vision is that the newly formed Committee may become the gateway to global and humanitarian activities and outreach for the EANS by creating and strengthening partnerships with other actors to address issues of surgical practice, education, research, advocacy and leadership. This, while maintaining and safeguarding high standards of clinical care, accessibility, safety and professionalism. The goals of the Committee are to enhance the Association's engagement with realities in need, to contribute to improving Global Health and Global Neurosurgery, to develop self-sustaining neurosurgical services in LMICs, and to provide a high level of care, curricula, education, certification and expertise.

Finally, the present survey collaterally allowed the creation of a list of over 200 EANS professionals that share the same vision of the Committee. According to this and all the results of this study, the EANS community seems ready to make its contribution to Global Neurosurgery advances.

## 5 Limitations

Although most of the EANS nations were included in this study, the results may not be representative of the entire community of European neurosurgeons. A possible bias common to all surveys with optional participation is that the responders may have a higher interest in the examined topic when compared to non-responders. This may have led to an over-estimation of the degree of interest in Global Neurosurgery in our sample. However, the survey was designed to examine interest and participation whereas a quantitative analysis of the actual Global Neurosurgery activities goes beyond the scopes of the study.

## 6 Conclusions

The degree of awareness and participation in Global Neurosurgery initiatives amongst the EANS community seems limited, although with some differences amongst professional categories. However, most remain interested in Global Neurosurgery and wish to have a positive impact in LMICs, while recognising Global Neurosurgery as a valuable career track. Most would accept to self-fund their rotation in a LMIC and most would be available to participate during their personal vacation time.

However, most training programs or job environments do not seem to encourage participation in Global Neurosurgery and faculty members with an interest in this topic are only present in a minority of cases. Several factors may hinder the growth of this subject amongst EANS members, and the addition of dedicated time, centralisation of information and the search for funding for these activities are desirable.

## Declaration of competing interest

The authors declare that they have no known competing financial interests or personal relationships that could have appeared to influence the work reported in this paper.
