# Supplementary material for: Global neurosurgery amongst the EANS community: Where are we at?
Source: Brain Spine. 2022 Jun 28;2:100911. doi: 10.1016/j.bas.2022.100911 (PMC9559959; doi:10.1016/j.bas.2022.100911)
Supplement: Multimedia component 1 [file mmc1.docx]

**Demographics**

| 1. **Age** |  |  |
| --- | --- | --- |
| **Choices** | **Number** | **%** |
| <25 | 3 | 0,9 |
| 25-29 | 55 | 16,6 |
| 30-34 | 142 | 42,9 |
| 35-39 | 58 | 17,5 |
| 50-59 | 68 | 20,5 |
| >60 | 5 | 1,5 |

| 1. **Birthplace** |  |  |
| --- | --- | --- |
| **Choices** | **Number** | **%** |
| Italy | 54 | 16,3 |
| Germany | 44 | 13,3 |
| Out of the EANS community | 26 | 7,9 |
| Spain | 23 | 6,9 |
| Portugal | 22 | 6,6 |
| Romania | 16 | 4,8 |
| Belgium | 13 | 3,9 |
| Greece | 12 | 3,6 |
| The Nederlands | 10 | 3,0 |
| Poland | 9 | 2,7 |
| Russia | 9 | 2,7 |
| Czech Republic | 7 | 2,1 |
| Switzerland | 7 | 2,1 |
| United Kingdom | 7 | 2,1 |
| Austria | 6 | 1,8 |
| Bulgaria | 6 | 1,8 |
| Hungary | 5 | 1,5 |
| Israel | 5 | 1,5 |
| Serbia | 5 | 1,5 |
| Turkey | 5 | 1,5 |
| Ukraine | 5 | 1,5 |
| Croatia | 4 | 1,2 |
| Estonia | 4 | 1,2 |
| Moldova | 4 | 1,2 |
| France | 3 | 0,9 |
| Lithuania | 3 | 0,9 |
| Slovenia | 3 | 0,9 |
| Sweden | 3 | 0,9 |
| Cyprus | 2 | 0,6 |
| Slovakia | 2 | 0,6 |
| Armenia | 1 | 0,3 |
| Bosnia & Herzegovina | 1 | 0,3 |
| Denmark | 1 | 0,3 |
| Finland | 1 | 0,3 |
| Kazakhstan | 1 | 0,3 |
| North Macedonia | 1 | 0,3 |
| Norway | 1 | 0,3 |

| 1. **EANS membership** |  |  |
| --- | --- | --- |
| **Choices** | **Number** | **%** |
| Yes | 255 | 77 |
| No | 76 | 23 |

| 1. **Marital status** |  |  |
| --- | --- | --- |
| **Choices** | **Number** | **%** |
| Married/engaged in a relationship | 215 | 65 |
| Single | 112 | 33,8 |
| Not answering | 4 | 1,2 |

| 1. **What is your current surgical status?** |  |  |
| --- | --- | --- |
| **Choices** | **Number** | **%** |
| Junior trainee | 47 | 14,2 |
| Senior trainee | 122 | 36,9 |
| Fellow | 23 | 6,9 |
| Specialist | 133 | 40,2 |

| 1. **What is the country where you are currently enrolled in a Neurosurgery Program or where did you complete your training in Neurosurgery?** |  |  |
| --- | --- | --- |
| **Choices** | **Number** | **%** |
| Germany | 70 | 21,1 |
| Italy | 44 | 13,3 |
| Spain | 27 | 8,2 |
| Portugal | 20 | 6,0 |
| United Kingdom | 16 | 4,8 |
| Switzerland | 13 | 3,9 |
| Belgium | 12 | 3,6 |
| Russia | 11 | 3,3 |
| France | 10 | 3,0 |
| Romania | 10 | 3,0 |
| Greece | 8 | 2,4 |
| Israel | 8 | 2,4 |
| Serbia | 8 | 2,4 |
| The Nederlands | 8 | 2,4 |
| Czech Republic | 7 | 2,1 |
| Hungary | 7 | 2,1 |
| Poland | 7 | 2,1 |
| Turkey | 5 | 1,5 |
| Ukraine | 5 | 1,5 |
| Austria | 4 | 1,2 |
| Croatia | 4 | 1,2 |
| Estonia | 4 | 1,2 |
| Finland | 4 | 1,2 |
| Sweden | 4 | 1,2 |
| Bulgaria | 3 | 0,9 |
| Moldova | 3 | 0,9 |
| Slovenia | 3 | 0,9 |
| Armenia | 1 | 0,3 |
| Denmark | 1 | 0,3 |
| Kazakhstan | 1 | 0,3 |
| Lithuania | 1 | 0,3 |
| North Macedonia | 1 | 0,3 |
| Norway | 1 | 0,3 |

| 1. **In which country are you currently practicing?** |  |  |
| --- | --- | --- |
| **Choices** | **Number** | **%** |
| Germany | 65 | 19,6 |
| Italy | 40 | 12,1 |
| Spain | 27 | 8,2 |
| Portugal | 22 | 6,6 |
| United Kingdom | 20 | 6,0 |
| France | 14 | 4,2 |
| Switzerland | 12 | 3,6 |
| Belgium | 12 | 3,6 |
| The Nederlands | 8 | 2,4 |
| Russia | 8 | 2,4 |
| Romania | 7 | 2,1 |
| Poland | 7 | 2,1 |
| Not answering | 7 | 2,1 |
| Hungary | 7 | 2,1 |
| Greece | 7 | 2,1 |
| Czech Republic | 7 | 2,1 |
| Out of EANS community | 6 | 1,8 |
| Israel | 6 | 1,8 |
| Ukraine | 5 | 1,5 |
| Turkey | 5 | 1,5 |
| Austria | 5 | 1,5 |
| Sweden | 4 | 1,2 |
| Serbia | 4 | 1,2 |
| Estonia | 4 | 1,2 |
| Croatia | 4 | 1,2 |
| Slovenia | 3 | 0,9 |
| Moldova | 3 | 0,9 |
| Bulgaria | 3 | 0,9 |
| Norway | 2 | 0,6 |
| Finland | 2 | 0,6 |
| North Macedonia | 1 | 0,3 |
| Lithuania | 1 | 0,3 |
| Kazakhstan | 1 | 0,3 |
| Denmark | 1 | 0,3 |
| Armenia | 1 | 0,3 |

| 1. **What is the population that your Institution serves?** |  |  |
| --- | --- | --- |
| **Choices** | **Number** | **%** |
| <1 million | 144 | 43,5 |
| 1-5 million | 19 | 5,7 |
| >5 million | 168 | 50,8 |

| 1. **During your medical school, did you spend at least an academical educational/clinical/research period abroad (Erasmus program, bilateral agreements between Universities…?)** |  |  |
| --- | --- | --- |
| **Choices** | **Number** | **%** |
| Yes | 198 | 59,8 |
| No | 133 | 40,2 |

| 1. **If yes, in which of the following World Bank regions? (more than one option available)** |  |  |
| --- | --- | --- |
| **Choices** | **Number** | **% (198)** |
| High Income (North America, Japan, Taiwan, Australia, New Zeland, and European countries) | 149 | 75,3 |
| Europe and Central Asia (excluding HICs) | 44 | 22,2 |
| Sub-Saharan Africa | 17 | 8,6 |
| Latin America and Caribbean | 13 | 6,6 |
| East Asia and Pacific | 9 | 4,5 |
| Middle East and North Africa | 6 | 3,0 |
| South Asia | 5 | 2,5 |

| 1. **If yes, how long was the total amount of time spent abroad during these experiences?** |  |  |
| --- | --- | --- |
| **Choices** | **Number** | **% (198)** |
| 1-2 weeks | 4 | 2,0 |
| 2-4 weeks | 16 | 8,1 |
| 1-6 months | 110 | 55,6 |
| >6 months | 68 | 34,3 |

| 1. **During your medical school, did you spend any extra-academical educational/clinical/research period abroad (volunteering, social programs…?)** |  |  |
| --- | --- | --- |
| **Choices** | **Number** | **%** |
| Yes | 129 | 39 |
| No | 202 | 61 |

| 1. **If yes, in which of the following World Bank regions? (more than one option available)** |  |  |
| --- | --- | --- |
| **Choices** | **Number** | **% (129)** |
| High Income (North America, Japan, Taiwan, Australia, New Zeland, and European countries) | 66 | 51,2 |
| Europe and Central Asia (excluding HICs) | 37 | 28,7 |
| Sub-Saharan Africa | 11 | 8,5 |
| Latin America and Caribbean | 13 | 10,1 |
| East Asia and Pacific | 7 | 5,4 |
| Middle East and North Africa | 7 | 5,4 |
| South Asia | 3 | 2,3 |

| 1. **If yes, how long was the total amount of time spent abroad during these experiences?** |  |  |
| --- | --- | --- |
| **Choices** | **Number** | **% (129)** |
| 1-2 weeks | 7 | 5,4 |
| 2-4 weeks | 45 | 34,9 |
| 1-6 months | 55 | 42,6 |
| >6 months | 17 | 13,2 |
| Not answering | 5 | 3,9 |

| 1. **I believe increasing access to neurosurgical care in low and middle income countries is a priority I will focus on in my career** |  |  |
| --- | --- | --- |
| **Choices** | **Number** | **%** |
| Strongly Agree | 100 | 30,2 |
| Agree | 112 | 33,8 |
| Neutral | 84 | 25,4 |
| Disagree | 29 | 8,8 |
| Strongly disagree | 6 | 1,8 |

| 1. **I believe global neurosurgery should be a valued and recognized possible career track (eg. compared with neurosurgeon-educator, neurosurgeon-scientist)** |  |  |
| --- | --- | --- |
| **Choices** | **Number** | **%** |
| Strongly Agree | 126 | 38,1 |
| Agree | 148 | 44,7 |
| Neutral | 49 | 14,8 |
| Disagree | 7 | 2,1 |
| Strongly disagree | 1 | 0,3 |

| 1. **I believe that residents in neurosurgery/neurosurgeons can have a positive local impact during rotations abroad** |  |  |
| --- | --- | --- |
| **Choices** | **Number** | **%** |
| Strongly Agree | 171 | 51,7 |
| Agree | 139 | 42,0 |
| Neutral | 18 | 5,4 |
| Disagree | 2 | 0,6 |
| Strongly disagree | 1 | 0,3 |

| 1. **If you are a trainee (or if you are not, when you were a trainee), does (did) your Residency Program offer(ed) possible international opportunities in low-middle income countries?** |  |  |
| --- | --- | --- |
| **Choices** | **Number** | **%** |
| Yes | 61 | 18,7 |
| No | 233 | 70,4 |
| I don’t know | 37 | 11,2 |
|  |  |  |
| 1. **Have you participated in a Global Neurosurgery rotation during your Residency?** |  |  |
| **Choices** | **Number** | **%** |
| Yes | 36 | 10,9 |
| No | 295 | 89,1 |

| 1. **If yes, in which of the following World Bank regions? (more than one option available)** |  |  |
| --- | --- | --- |
| **Choices** | **Number** | **% (36)** |
| High Income (North America, Japan, Taiwan, Australia, New Zeland, and European countries) | 20 | 51,2 |
| Europe and Central Asia (excluding HICs) | 3 | 28,7 |
| Sub-Saharan Africa | 3 | 8,5 |
| Latin America and Caribbean | 2 | 10,1 |
| East Asia and Pacific | 10 | 5,4 |
| Middle East and North Africa | 6 | 5,4 |

| 1. **If yes, how long was the total amount of time spent abroad during these experiences?** |  |  |
| --- | --- | --- |
| **Choices** | **Number** | **% (36)** |
| 1-2 weeks | 3 | 8,3 |
| 2-4 weeks | 3 | 8,3 |
| 1-6 months | 18 | 50,0 |
| >6 months | 10 | 27,8 |
| Not answering | 2 | 5,6 |

| 1. **Participation in a Global Neurosurgery rotation by my residency program/job environment is:** |  |  |
| --- | --- | --- |
| **Choices** | **Number** | **%** |
| Strongly encouraged | 44 | 13,3 |
| Encouraged | 42 | 12,7 |
| Neutral | 186 | 56,2 |
| Discouraged | 42 | 12,7 |
| Strongly discouraged | 17 | 5,1 |

| 1. **Are there any members in your Department involved in Global Neurosurgery projects?** |  |  |
| --- | --- | --- |
| **Choices** | **Number** | **%** |
| Yes | 77 | 23,3 |
| No | 209 | 63,1 |
| I don’t know | 45 | 13,6 |
| 1. **I am aware of Global Neurosurgery opportunities for residents/neurosurgeons** |  |  |
| **Choices** | **Number** | **%** |
| Yes | 125 | 38,1 |
| No | 206 | 62,2 |

| 1. **Do you follow Global Neurosurgery developments and updates?** |  |  |
| --- | --- | --- |
| **Choices** | **Number** | **%** |
| Yes | 123 | 37,2 |
| No | 208 | 62,8 |

| 1. **I am interested in participating in a Global Neurosurgery rotation during my residency/job** |  |  |
| --- | --- | --- |
| **Choices** | **Number** | **%** |
| Strongly Agree | 155 | 46,8 |
| Agree | 125 | 37,8 |
| Neutral | 42 | 12,7 |
| Disagree | 7 | 2,1 |
| Strongly disagree | 2 | 0,6 |

| 1. **I plan to participate in a Global Neurosurgery rotation as a neurosurgeon** |  |  |
| --- | --- | --- |
| **Choices** | **Number** | **%** |
| Strongly Agree | 116 | 35,0 |
| Agree | 94 | 28,4 |
| Neutral | 98 | 29,6 |
| Disagree | 17 | 5,1 |
| Strongly disagree | 6 | 1,8 |

| 1. **I would use my vacation to participate in a Global Neurosurgery rotation** |  |  |
| --- | --- | --- |
| **Choices** | **Number** | **%** |
| Yes | 168 | 50,8 |
| No | 49 | 14,8 |
| Maybe | 114 | 34,4 |

| 1. **I would be prepared to personally fund my participation in a Global Neurosurgery rotation** |  |  |
| --- | --- | --- |
| **Choices** | **Number** | **%** |
| Yes, completely | 78 | 23,6 |
| Yes, partially | 186 | 56,2 |
| No | 67 | 20,2 |

| 1. **Estimated ideal duration of a Global Neurosurgery rotation** |  |  |
| --- | --- | --- |
| **Choices** | **Number** | **%** |
| 1-2 weeks | 36 | 10,9 |
| 2-4 weeks | 103 | 31,1 |
| 1-6 months | 162 | 48,9 |
| >6 months | 20 | 6,0 |
| Not answering | 10 | 3,0 |

| 1. **If yes, in which of the following World Bank regions? (more than one option available)** |  |  |
| --- | --- | --- |
| **Choices** | **Number** | **%** |
| High Income (North America, Japan, Taiwan, Australia, New Zeland, and European countries) | 168 | 50,8 |
| Europe and Central Asia (excluding HICs) | 149 | 45,0 |
| Sub-Saharan Africa | 155 | 46,8 |
| Latin America and Caribbean | 173 | 52,3 |
| East Asia and Pacific | 173 | 52,3 |
| Middle East and North Africa | 129 | 39,0 |
| Not answering | 10 | 3,0 |

| 1. **Why would you choose the World Bank Regions declared in the previous question? (more than one option)** |  |  |
| --- | --- | --- |
| **Choices** | **Number** | **%** |
| Clinical (non-surgical) exposure | 102 | 30,8 |
| Higher surgical exposure | 209 | 63,1 |
| Better cultural experience | 165 | 49,8 |
| Higher level of neurosurgical unmed need | 156 | 47,1 |
| Better research opportunities | 88 | 26,6 |
| The region would be safer than others | 80 | 24,2 |
| Not answering | 10 | 3,0 |

| 1. **If a structured program was available through my Residency Program/job, my degree of interest in participation would be:** |  |  |  |  |
| --- | --- | --- | --- | --- |
| **Choices** | **Number** | **%** |  |  |
| Very interested | 183 | 55,3 |  |  |
| Interested | 119 | 36,0 |  |  |
| Neutral | 25 | 7,6 |  |  |
| Uninterested | 4 | 1,2 |  |  |
| Very uninterested | 0 | 0,0 |  |  |
| 1. **Rate from 0 to 3 (0=no important, 3 very important) each possible factor that would increase your participation (more than one option)** | **0** | **1** | **2** | **3** |
| **Choices** | **N (%)** | **N (%)** | **N (%)** | **N (%)** |
| Funding opportunities | 18 (5,4) | 51 (15,4) | 115 (34,7) | 147 (44,4) |
| Involvement of a staff neurosurgeon from my Institution | 64 (19,3) | 89 (26,9) | 92 (27,8) | 86 (26) |
| Residency/job requirement | 35 (10,6) | 92 (27,8) | 112 (33,8) | 92 (27,8) |
| Dedicated elective time for participation | 12 (3,6) | 58 (17,5) | 135 (40,8) | 126 (38,1) |

| 1. **I am most interested in participating in a Global Neurosurgery rotation with a role in (more than one option)** |  |  |
| --- | --- | --- |
| **Choices** | **Number** | **%** |
| Observation | 81 | 24,5 |
| Teaching | 163 | 49,2 |
| Clinical with no surgical exposure | 32 | 9,7 |
| Clinical with surgical exposure | 296 | 89,4 |
| Research | 111 | 33,5 |
| Not answering | 6 | 1,8 |

| 1. **Rate from 0 to 3 (0=no important, 3 very important) each possible motivating factors for participating in Global Neurosurgery rotation (more than one option)** | **0** | **1** | **2** | **3** |
| --- | --- | --- | --- | --- |
| **Choices** | **N (%)** | **N (%)** | **N (%)** | **N (%)** |
| Cultural experience | 9 (2,7) | 43 (13) | 128 (38,7) | 151 (45,6) |
| Altruistic goal | 17 (5,1) | 72 (21,8) | 112 (33,8) | 130 (39,3) |
| Enhancing clinical/technical exposure | 16 (4,8) | 46 (13,9) | 114 (34,4) | 155 (46,8) |
| Personal goal | 23 (6,9) | 69 (20,8) | 129 (39) | 110 (33,2) |
| Building international contacts | 10 (3) | 59 (17,8) | 144 (43,5) | 118 (35,6) |
| Enhancing CV | 58 (17,5) | 114 (34,4) | 102 (30,8) | 57 (17,2) |
| Language learning | 55 (16,6) | 97 (29,3) | 106 (32) | 73 (22,1) |

| 1. **Rate from 0 to 3 (0=no important, 3= very important) each possible barrier limiting participation in a Global Neurosurgery rotation** | **0** | **1** | **2** | **3** |
| --- | --- | --- | --- | --- |
| **Choices** | **N (%)** | **N (%)** | **N (%)** | **N (%)** |
| Lack of time | 10 (3) | 66 (19,9) | 119 (36) | 136 (41,1) |
| Financial concerns | 19 (5,7) | 98 (29,6) | 108 (32,6) | 106 (32) |
| Fear of missing training | 85 (25,7) | 115 (34,7) | 81 (24,5) | 50 (15,1) |
| Family/social limitations | 54 (16,3) | 104 (31,4) | 92 (27,8) | 81 (24,5) |
| Lack of program support | 14 (4,2) | 96 (29) | 119 (36) | 102 (30,8) |
| Fear of missing electives for fellowship | 75 (22,7) | 118 (35,6) | 93 (28,1) | 45 (13,6) |
| Lack of available opportunities | 27 (8,2) | 88 (26,6) | 122 (36,9) | 94 (28,4) |
| Program not willing to give time/remove from call schedule | 33 (10) | 118 (35,6) | 115 (34,7) | 65 (19,6) |
| Hospital conditions | 56 (16,9) | 137 (41,4) | 105 (31,7) | 33 (10) |
| Living conditions | 65 (19,6) | 135 (40,8) | 95 (28,7) | 36 (10,9) |
| Personal safety | 47 (14,2) | 111 (33,5) | 113 (34,1) | 60 (18,1) |
| Fear of inexperience | 87 (26,3) | 134 (40,5) | 85 (25,7) | 25 (7,6) |
| Fear of litigation | 88 (26,6) | 145 (43,8) | 74 (22,4) | 24 (7,3) |
